# PET/CT Imaging as a Diagnostic Tool in Distinguishing Well-Differentiated versus Dedifferentiated Liposarcoma

**DOI:** 10.1155/2020/8363986

**Published:** 2020-05-29

**Authors:** Amanda Parkes, Elizabeth Urquiola, Priya Bhosale, Heather Lin, Kelsey Watson, Wei-Lien Wang, Barry Feig, Keila Torres, Christina L. Roland, Anthony P. Conley, Maria Zarzour, J. Andrew Livingston, Ravin Ratan, Joseph Ludwig, Dejka M. Araujo, Vinod Ravi, Robert S. Benjamin, Shreyaskumar Patel, Neeta Somaiah

**Affiliations:** ^1^University of Wisconsin Carbone Cancer Center, Madison, WI, USA; ^2^The University of Texas MD Anderson Cancer Center, Division of Cancer Medicine, Houston, TX, USA; ^3^The University of Texas MD Anderson Cancer Center, Department of Sarcoma Medical Oncology, Houston, TX, USA; ^4^The University of Texas MD Anderson Cancer Center, Department of Diagnostic Radiology, Houston, TX, USA; ^5^The University of Texas MD Anderson Cancer Center, Department of Biostatistics, Houston, TX, USA; ^6^The University of Texas MD Anderson Cancer Center, Department of Surgical Oncology, Houston, TX, USA; ^7^The University of Texas MD Anderson Cancer Center, Department of Pathology, Houston, TX, USA

## Abstract

Distinguishing well-differentiated liposarcoma (WDLPS) from dedifferentiated liposarcoma (DDLPS) is essential given distinct treatment paradigms and chemosensitivity. Percutaneous biopsy has a low sensitivity for detecting DDLPS. We sought to identify the diagnostic utility of positron emission tomography/computed tomography (PET/CT) in identifying WDLPS versus DDLPS. An independent radiologist reviewed PET/CT images to identify target lesions and determine the maximum standardized uptake value (SUVmax). An independent pathologist review confirmed WDLPS or DDLPS histology. A binary cutoff point of SUVmax was identified using a classification and regression trees (CART) algorithm. We identified 20 patients with WDLPS or DDLPS with 26 PET/CTs performed for separate recurrences that were followed by surgical sampling. Of the 26 records, 12 were DDLPS (46%) and 14 were WDLPS (54%). Patients with DDLPS had significantly higher SUVmax than those with WDLPS (*p* value = 0.0035). A SUVmax of 4 was identified as the cutoff point. Using this cutoff, the sensitivity of SUVmax identifying a case as DDLPS was 83.3% (95% CI: 51.6%, 97.9%) and the specificity was 85.7% (95% CI: 57.2%, 98.2%). PET/CT is a sensitive and specific diagnostic tool to identify the presence of dedifferentiation within the tumor.

## 1. Introduction

Liposarcomas are malignant tumors of adipocytic differentiation that are categorized into several subtypes with diverse biology, clinical behavior, and treatment approaches. Together, well-differentiated liposarcoma (WDLPS) and dedifferentiated liposarcoma (DDLPS) represent the majority of all liposarcomas, with WDLPS representing approximately 40–50% of all liposarcomas and DDLPS representing 15–20% [1]. WDLPS and DDLPS are the predominant sarcoma subtype in the retroperitoneum and usually present as large tumors occupying most of the abdominal or pelvic cavity but can also be multifocal. WDLPS and DDLPS often coexist as heterogeneous tumors, but DDLPS can appear during progression or recurrence of a WDLPS. Prognosis is worse with presence of a dedifferentiated/high-grade component within the tumor, with a 2003 study by Singer et al. showing a sixfold increased risk of death with DDLPS versus WDLPS [2]. Since the presence of dedifferentiation drives the prognosis, tumors with a component of dedifferentiation are labeled DDLPS.

Surgery is the primary treatment for WDLPS and DDLPS; however, rate of local recurrence can be as high as 80% at 5 years [3]. The tumors can invade surrounding structures making repeated surgeries challenging, adding to morbidity and mortality. Aggressive surgery with resection of adjoining organs has not shown significant survival benefit, and hence, each case should be individually evaluated to determine the extent of surgery [4]. Chemotherapy can be beneficial in DDLPS, with a study by Livingston et al. showing 21% of DDLPS patients having a partial response and 40% having stable disease with chemotherapy by Response Evaluation Criteria In Solid Tumors (RECIST). However, chemotherapy does not benefit purely WDLPS [5, 6]. As noted in this paper, given the large and heterogeneous nature of these tumors, response is often manifested as a decrease in vascularity without significant shrinkage in size, making response assessment via RECIST challenging in these patients. Given the poor prognosis in DDLPS patients and the challenges with repeated surgeries, a multidisciplinary approach is often adopted with consideration given to neoadjuvant chemotherapy in locally recurrent/locally advanced DDLPS. Given the significant differences in prognosis, clinical behavior, and treatment approaches to WDLPS and DDLPS, it is critical to accurately identify DDLPS prior to initiation of treatment.

As these tumors can be large and heterogeneous, a biopsy can often miss the high-grade DDLPS area within the tumor. Ikoma et al. showed that percutaneous biopsy has a sensitivity of only 36% for detecting DDLPS [7]. Even though computed tomography (CT) characteristics are often helpful in differentiating WDLPS from DDLPS, this is not always obvious, and frequently, the pathology at surgery does not match what was expected based on CT imaging and biopsy. Studies have shown high sensitivity of CT to identify DDLPS (>90%), but relatively poor specificity (52%), highlighting that high-density areas on CT do not always correlate with dedifferentiation [8, 9]. Given the diagnostic challenges faced in the accurate identification of DDLPS that have direct impact on treatment decisions, we conducted a study to determine the utility of positron emission tomography/CT (PET/CT) in identifying areas of dedifferentiation in the tumor. The importance of accurate identification of DDLPS is even more relevant as newer therapeutics directed at DDLPS are entering the clinical arena. A pathologic confirmation of dedifferentiation is often required and knowing which part of the tumor to direct the biopsy needle could significantly increase the sensitivity and specificity of the biopsy. Given these important considerations, we sought to identify the diagnostic utility of PET/CT in identifying WDLPS versus DDLPS. The working hypothesis for this study was that PET/CT would have a higher sensitivity and specificity for detection of DDLPS than that seen previously with CT-guided percutaneous biopsy.

## 2. Materials and Methods

WDLPS and DDLPS patients seen at the University of Texas MD Anderson Cancer Center between March 2005 and March 2015 were identified via the institutional tumor registry and surgical database. We retrospectively reviewed the charts of all WDLPS and DDLPS patients to identify those who had PET/CT imaging anytime during their disease course in our system. We recorded demographic data including gender, ethnicity, and age at diagnosis. The study was conducted in accordance with all relevant guidelines and procedures and approved by the University of Texas MD Anderson Cancer Center Institutional Review Board. Informed consent requirement was waived given the retrospective design of the study.

PET/CT imaging was performed using the standard protocol. Fasting for 6 hours was required prior to F-18 FDG administration to achieve a blood glucose level of less than 120 mg/dL. Intravenous F-18 FDG (185–370 MBq/injection; 5–10 mCi/injection) was administered and the patients then rested in a quiet room. PET/CT images were acquired 60 minutes after F-18 FDG administration. Images were acquired on a Discovery ST, STE, or RX; GE Healthcare platform. Standard vendor-provided reconstruction algorithms were used to reconstruct the PET images. Non-contrast-enhanced CT images were from the base of the skull to proximal thighs at a 3.75 mm slice thickness. Attenuation and nonattenuation-corrected datasets were reconstructed, and the images were analyzed on a MIM vista work station.

In order to assess for meaningful differences in SUVmax of target lesions for DDLPS and WDLPS, we included only those patients who had a PET/CT with no therapy in the preceding three months prior to the PET/CT. Patients were also required to have definite pathologic confirmation of WDLPS or DDLPS. The preferred method of pathologic confirmation was post-PET/CT surgical resection without intervening treatment/intervention between PET/CT and time of surgical resection, which was considered as the gold standard in this analysis. In patients without post-PET/CT surgical resection, biopsy was allowed as pathologic confirmation if biopsy was performed within 60 days of PET/CT and showed DDLPS. Biopsy showing only WDLPS was not included as definite pathologic confirmation, given we could not exclude focus of dedifferentiation [7]. Pathology describing WDLPS with focal DDLPS was considered DDLPS for this analysis. Multiple PET/CTs from the same patient were only included if different or new lesions/recurrences were identified and analyzed at different time points. An independent radiologist reviewed PET/CT images to identify target lesions and determine the SUVmax. An independent pathologist reviewed archived samples to confirm WDLPS or DDLPS histology. Analyses were conducted using both all records per patient as well as only one record per patient. For analyses using only one record per patient, a random record of each patient was chosen.

The distribution of SUVmax was summarized by BLiP plots [10]. The Wilcoxon rank-sum test was used to compare the difference of SUVmax between the two different pathologic diagnoses [11]. A logistic regression model [12] was used to assess the ability of SUVmax in predicting pathologic diagnosis. The transformation of logarithm to the base 2 of SUVmax was used in this analysis to reduce the influences of outliers. The binary cutoff point of SUVmax was identified using a CART algorithm in which a cutoff point is determined for each predictor variable such that two resulting subgroups are the most different in their outcome. Sensitivity and specificity of the cutoff was calculated along with 95% confidence interval (CI).

## 3. Results

We identified 1194 patients from our institutional registry with liposarcoma including five with head and neck primaries, 840 with extremity primaries, and 349 patients with retroperitoneal primaries. Of these patients, 20 patients with WDLPS or DDLPS were identified as having PET/CTs (26 individual scans for separate recurrences) that met our inclusion criteria. The majority of pathologic confirmations following PET/CT were via surgical resection (21/26, 81%), with only 5 PET/CTs included with post-PET/CT pathologic confirmation by biopsy showing DDLPS.

An additional 10 patients were identified with 16 PET/CTs performed for separate individual recurrences of WDLPS or DDLPS. These 16 records were excluded for the following reasons: six records had biopsy showing only WDLPS without surgical resection, one record was missing maximum standardized uptake value (SUVmax) confirmation given lack of available images for independent radiology review, eight records had no pathologic confirmation of diagnosis, and one record was excluded as it was the same lesion as one previously included and not a separate individual recurrence.

Of the 20 patients included in the study, 8 (40%) were female and 12 (60%) were male. Mean and median age were 60 and 64 years, respectively (range 29–81 years). The majority of patients were Caucasian (16/20, 80%), with two Hispanic patients (10%), one Asian patient (5%), and one African American patient (5%). Of the 20 patients, 11 patients (55%) had DDLPS and nine patients (45%) had WDLPS. There were only three patients with multiple PET/CTs performed for separate individual recurrences meeting our inclusion criteria (one patient with five records and two patients with two records each). Patient characteristics are detailed in Table 1.

**Figure 1:**
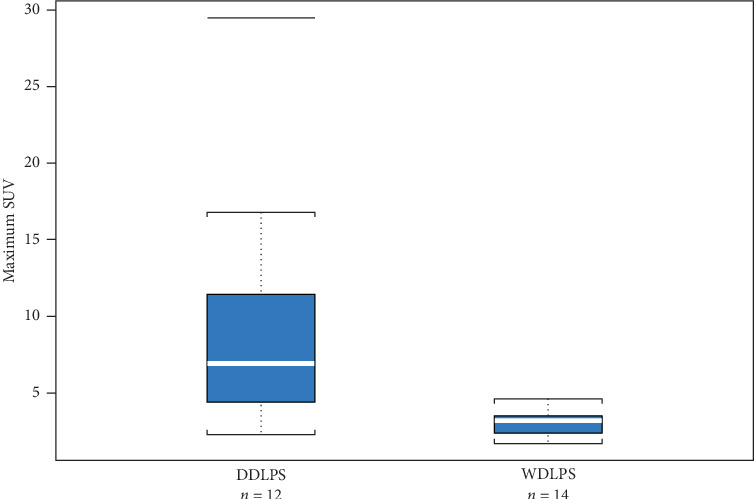
Distributions of SUVmax by pathologic diagnosis.

Including all 26 PET/CT records, 12 were for DDLPS (46%) and 14 were for WDLPS (54%). The SUVmax ranged from 1.7 to 29.5. Comparing SUVmax values for DDLPS to WDLPS, we found that patients with DDLPS had significantly higher SUVmax than those with WDLPS. Mean and median SUVmax for DDLPS were 9.23 and 6.9, respectively, as compared with 3.15 and 3.2, respectively, for WDLPS (*p* value = 0.0035, Table 2). The SUVmax range was 2.3–29.5 for DDLPS and 1.7–4.6 for WDLPS.  Figure 1 shows distributions of SUVmax by pathologic diagnosis.

Review of the three patients with two or more PET/CTs performed for separate recurrences followed by pathologic confirmation showed relative concordance between pathology and SUVmax. One patient had two PET/CTs done 5 months apart (SUVmax 2.4 and 3.3) with surgical resection following each PET/CT showing WDLPS. Another patient had two PET/CTs done 4 months apart (SUVmax 6.4 and 7.4) with biopsy following each PET/CT showing DDLPS. The final patient had 5 PET/CTs over the course of 5.5 years (SUVmax range 1.7–4.6), each followed by surgical resection showing WDLPS.

**Figure 2:**
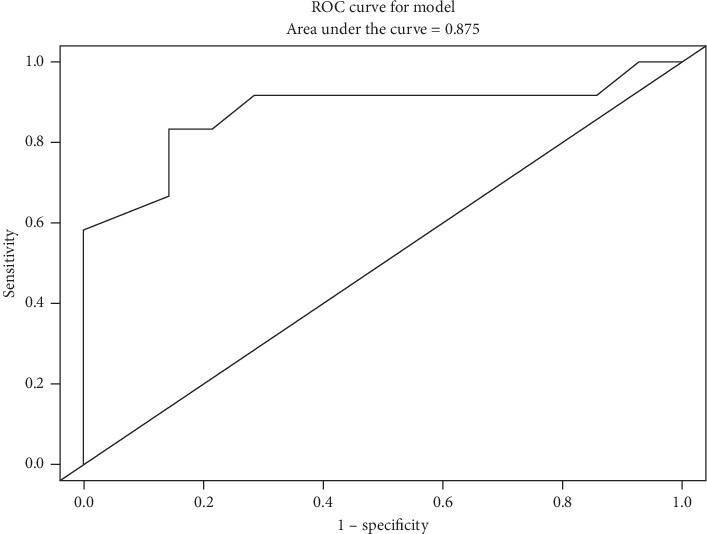
Receiver-operating characteristic (ROC) curve for SUVmax in discriminating DDLPS versus WDLPS.

When SUVmax, as the only independent variable, was included in a logistic regression model of pathologic diagnosis (DDLPS versus WDLPS) the area under the curve was 0.875 (95% CI: 0.719, 1.0, Figure 2). Each point on the receiver-operating characteristic (ROC) curve shown in Figure 2 represents a sensitivity/specificity pair corresponding to a particular decision threshold. A SUVmax of 4 was identified as the optimal cutoff point using the classification and regression trees (CART) algorithm. Using this cutoff, there were 14 patients with SUVmax less than 4, 12 with WDLPS (86%), and 2 with DDLPS (14%). Of the two patients with SUVmax less than 4 and DDLPS, SUVmax values were 2.3 and 3.5. There were 12 patients with SUVmax of 4 or greater, 2 with WDLPS (17%), and 10 with DDLPS (83%). Of the two patients with SUVmax of 4 or greater and WDLPS, SUVmax value was 4.6 in both patients. The sensitivity of SUVmax identifying a case as DDLPS was 83.3% (95% CI: 51.6%, 97.9%), and the specificity was 85.7% (95% CI: 57.2%, 98.2%).

The aforementioned analyses were also performed using only one record per patient, therefore including only 20 records. Using only 20 records from 20 patients, we again found that DDLPS had significant higher SUVmax than WDLPS with mean and median SUVmax in DDLPS of 9.49 and 7.4, respectively, as compared with 3.22 and 3.3, respectively, for WDLPS (*p* value = 0.0142). SUVmax range was 2.3–29.5 for DDLPS and 2.3–4.6 for WDLPS. Using only these 20 records, when SUVmax, as the only independent variable, was included in the logistic regression model of pathologic diagnosis (DDLPS versus WDLPS), the area under the curve was 0.864 (95% CI: 0.683, 1.0). A SUVmax of 4 was identified as the cutoff point. The sensitivity of SUVmax identifying a case as DDLPS was 81.8% (95% CI: 48.2%, 97.7%), and the specificity was 88.9% (95% CI: 51.8%, 99.7%).

## 4. Discussion

WDLPS and DDLPS constitute the most common liposarcoma subtypes and account for 11% of all soft tissue sarcomas. The ability to distinguish between WDLPS and DDLPS is important as these patients have distinct prognoses and outcomes to treatments, particularly chemotherapy. Given the heterogeneity of these tumors, surgical resection with pathologic review is the gold standard for identification of DDLPS. However, in the setting of unresectable disease or when neoadjuvant chemotherapy approaches are being utilized, it is essential to find a nonsurgical approach to differentiate between WDLPS and DDLPS. As previously mentioned, percutaneous biopsy has a low sensitivity for detecting DDLPS within these large, heterogeneous, and often multifocal tumors [7].

**Figure 3:**
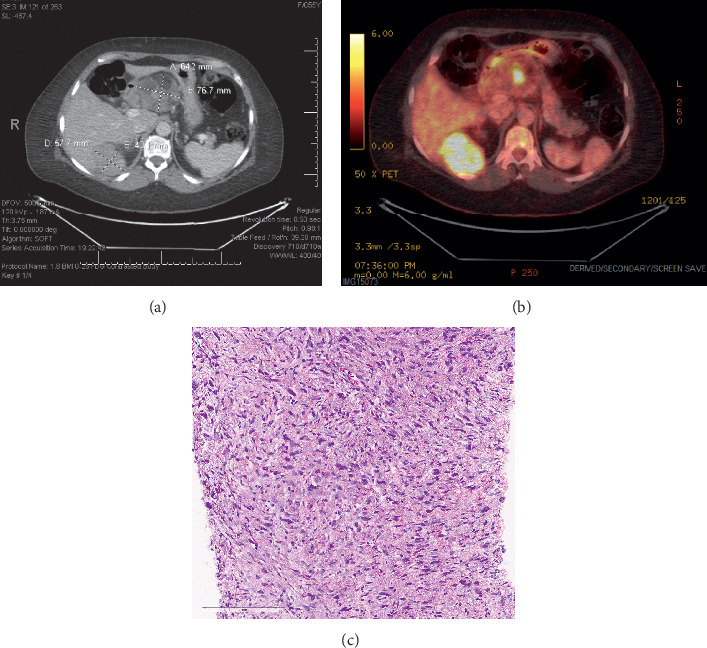
CT (a) versus PET (b) from a patient where the initial biopsy, done prior to the PET directed at the mass adjacent to the pancreas, showed WDLPS. The PET clearly shows only a small area within this mass to have FDG activity. Repeat biopsy from the FDG avid right perinephric mass demonstrated cellular nonlipogenic component with pleomorphic and atypical spindle cells and mitoses consistent with DDLPS (c).

Using 20 patients with pathologic confirmation of DDLPS or WDLPS who underwent PET/CT with out therapy in the preceding three months, we were able to show high sensitivity and specificity of identifying DDLPS using a PET/CT SUVmax cutoff of 4. We noted that higher SUVmax was associated with higher likelihood of having a focus of DDLPS (SUVmax range for WDLPS: 1.7–4.6 and SUVmax range for DDLPS: 2.3–29.5). The sensitivity of using SUVmax (81.8%) is much higher than the 36% sensitivity seen with percutaneous biopsy for the detection of DDLPS [7]. These data support evaluating the role of PET/CT in diagnostic strategies for liposarcoma patients. Challenging cases of large heterogeneous tumors can utilize PET/CT to provide guidance regarding presence of dedifferentiation and help select the site for biopsy. Illustratively, in our patient cohort, of the 21 patients who had pathologic confirmation by post-PET/CT surgical resection, 13 patients had a preceding biopsy prior to the PET/CT that allowed for comparison between biopsy and surgical resection pathology. Of these 13 patients, only two had evidence of DDLPS on biopsy, while five patients had DDLPS on surgical resection (3/5, 60% of the biopsies were false negative for DDLPS). The use of PET/CT prior to biopsy may facilitate improved diagnostic accuracy of percutaneous biopsy if directed to the area of highest SUV. As an illustrative example, one patient in our institutional database had a biopsy directed at the larger mass abutting the pancreatic head showing WDLPS, but a later PET/CT revealed that mass to only have a small area of nodular activity (Figure 3). The highest FDG avidity was noted in the mass posterior to the right renal pole with SUVmax 9.05, which was subsequently biopsy-proven DDLPS.

Limitations of this study include its retrospective nature and small sample size. The small sample size is due to the fact that PET/CT is not approved for this indication and hence not obtained as standard practice prior to surgery at our institution. Additionally, many of the patients with DDLPS or WDLPS who had PET/CT performed were excluded as they had intervening therapy prior to surgery or did not undergo surgery. The results, nevertheless, support further evaluation of the role of PET/CT to differentiate these tumors preoperatively. Incorporating PET/CT in future studies of DDLPS and WDLPS patients can help us better refine the diagnostic algorithms, further optimize/confirm the best SUVmax cutoff value, and it may also be a useful tool to identify tumor response to therapy in the future.

## 5. Conclusions

The ability to accurately identify dedifferentiated liposarcoma from purely well-differentiated liposarcoma upfront, prior to surgical resection, is critical, as it not only changes prognosis but can also alter the treatment approach. Given the high rate of recurrence, neoadjuvant chemotherapy is often considered in recurrent dedifferentiated liposarcoma, and occasionally for a primary tumor, especially if surgery might result in a nephrectomy. However, for purely well-differentiated liposarcoma, there is no role for neoadjuvant chemotherapy as it does not respond to current standard systemic therapies. Our current work shows that PET/CT has high sensitivity and specificity for identifying presence of dedifferentiated liposarcoma in the tumor, using a SUVmax cut-off of 4 to help differentiate between the two liposarcoma subtypes. We believe that these findings are of significant importance and that these data support inclusion of PET/CT in the initial diagnostic strategy for liposarcoma patients where the presence or absence of dedifferentiation can change the treatment decision. PET/CT can also be particularly helpful in guiding the location for biopsy in these heterogeneous tumors.

## Figures and Tables

**Table 1:** Patient characteristics of 20 WDLPS or DDLPS patients meeting inclusion criteria.

Patient characteristics (*n* = 20)	No. (%)
Median age	64 years (range 29–81 years)
Sex	
Female	8 (40%)
Male	12 (60%)
Ethnicity	
Caucasian	16 (80%)
Hispanic	2 (10%)
Asian	1 (5%)
African American	1 (5%)
Diagnosis	
DDLPS	11 (55%)
WDLPS	9 (45%)
Number of PET/CTs performed for separate individual recurrences	
One	17 (85%)
Two	2 (10%)
Five	1 (5%)

Characteristics for 26 PET/CTs meeting inclusion criteria (*n* = 26)	

Median time from PET to tissue collection in days	32.5 days
Method of pathologic confirmation	
Surgical resection	21 (81%)
Biopsy	5 (19%)

**Table 2:** Comparison of SUVmax values between WDLPS and DDLPS diagnoses.

Variable	Pathologic diagnosis	*N*	Mean	Standard deviation	Standard error	Minimum	Maximum	Median	First quartile	Third quartile	*p* value
SUVmax	DDLPS	12	9.23	7.63	2.20	2.3	29.5	6.9	4.4	11.45	0.0035

	WDLPS	14	3.15	0.84	0.22	1.7	4.6	3.2	2.4	3.50	

## Data Availability

The datasets generated during and/or analyzed during the current study are available from the corresponding author on reasonable request.

## References

[1] Lee A. T. J., Thway K., Huang P. H., Jones R. L. (2018). Clinical and molecular spectrum of liposarcoma. *Journal of Clinical Oncology*.

[2] Singer S., Antonescu C., Riedel E., Brennan M. F. (2003). Histologic subtype and margin of resection predict pattern of recurrence and survival for retroperitoneal liposarcoma. *Annals of Surgery*.

[3] Singer S., Corson J. M., Demetri G. D., Healey E. A., Marcus K., Eberlein T. J. (1995). Prognostic factors predictive of survival for truncal and retroperitoneal soft-tissue sarcoma. *Annals of Surgery*.

[4] Tseng W. W., Madewell J. E., Wei W. (2014). Locoregional disease patterns in well-differentiated and dedifferentiated retroperitoneal liposarcoma: implications for the extent of resection?. *Annals of Surgical Oncology*.

[5] Livingston J. A., Bugago D., Barbo A. (2017). Role of chemotherapy in dedifferentiated liposarcoma of the retroperitoneum: defining the benefit and challenges of the standard. *Scientific Reports*.

[6] Italiano A., Toulmonde M., Cioffi A. (2012). Advanced well-differentiated/dedifferentiated liposarcomas: role of chemotherapy and survival. *Annals of Oncology*.

[7] Ikoma N., Torres K. E., Somaiah N. (2015). Accuracy of preoperative percutaneous biopsy for the diagnosis of retroperitoneal liposarcoma subtypes. *Annals of Surgical Oncology*.

[8] Bhosale P., Wang J., Varma D. (2016). Can abdominal computed tomography imaging help accurately identify a dedifferentiated component in a well-differentiated liposarcoma?. *Journal of Computer Assisted Tomography*.

[9] Lahat G., Madewell J. E., Anaya D. A. (2009). Computed tomography scan-driven selection of treatment for retroperitoneal liposarcoma histologic subtypes. *Cancer*.

[10] Lee J. J., Tu Z. N. (1997). A versatile one-dimensional distribution plot: the BLiP plot. *The American Statistician*.

[11] Woolson R. F., Clarke W. R. (2000). *Statistical Methods for the Analysis of Biomedical Data*.

[12] Hosmer D. W., Lemeshow S. (2000). *Applied Logistic Regression*.

